# Interventions for increasing colorectal cancer screening uptake among African-American men: A systematic review and meta-analysis

**DOI:** 10.1371/journal.pone.0238354

**Published:** 2020-09-16

**Authors:** Charles R. Rogers, Phung Matthews, Lei Xu, Kenneth Boucher, Colin Riley, Matthew Huntington, Nathan Le Duc, Kola S. Okuyemi, Margaret J. Foster

**Affiliations:** 1 Department of Family & Preventive Medicine, University of Utah School of Medicine, Salt Lake City, Utah, United States of America; 2 Department of Health Education and Promotion, East Carolina University, Greenville, NC, United States of America; 3 Cancer Biostatistics Shared Resource, Huntsman Cancer Institute, Salt Lake City, UT, United States of America; 4 Medical Sciences Library, Texas A&M University, College Station, TX, United States of America; Georgia Southern University, UNITED STATES

## Abstract

**Background:**

African-American men have the lowest 5-year survival rate in the U.S. for colorectal cancer (CRC) of any racial group, which may partly stem from low screening adherence. It is imperative to synthesize the literature evaluating the effectiveness of interventions on CRC screening uptake in this population.

**Materials and methods:**

In this systematic review and meta-analysis, Medline, CINAHL, Embase, and Cochrane CENTRAL were searched for U.S.-based interventions that: were published after 1998–January 2020; included African-American men; and evaluated CRC screening uptake explicitly. Checklist by Cochrane Collaboration and Joanna Brigg were utilized to assess risk of bias, and meta-regression and sensitivity analyses were employed to identify the most effective interventions.

**Results:**

Our final sample comprised 41 studies with 2 focused exclusively on African-American men. The most frequently adopted interventions were educational materials (39%), stool-based screening kits (14%), and patient navigation (11%). Most randomized controlled trials failed to provide details about the blinding of the participant recruitment method, allocation concealment method, and/or the outcome assessment. Due to high heterogeneity, meta-analysis was conducted among 17 eligible studies. Interventions utilizing stool-based kits or patient navigation were most effective at increasing CRC screening completion, with odds ratios of 9.60 (95% CI 2.89–31.82, p = 0.0002) and 2.84 (95% CI 1.23–6.49, p = 0.01). No evidence of publication bias was present for this study registered with the International Prospective Registry of Systematic Reviews (PROSPERO 2019 CRD42019119510).

**Conclusions:**

Additional research is warranted to uncover effective, affordable interventions focused on increasing CRC screening completion among African-American men. When designing and implementing future multicomponent interventions, employing 4 or fewer interventions types may reduce bias risk. Since only 5% of the interventions solely focused on African-American men, future theory-driven interventions should consider recruiting samples comprised solely of this population.

## Introduction

African-American men and women in the United States (U.S.) have the highest rates of most cancers in terms of both mortality and morbidity [1]. Despite being highly treatable when detected early, colorectal cancer (CRC) remains the second leading cause of death in the U.S. from cancers affecting both men and women, and the third deadliest among African Americans [2]. African-American men are disproportionately affected by CRC, experiencing the lowest 5-year survival rate of all racial and gender groups. Furthermore, African-American men are 24% more likely to have CRC than white men [3]. Contributing factors to these CRC incidence and mortality inequities among African-American men include a lack of health insurance and limited access to early detection screening, in addition to socioeconomic disadvantages such as lower education levels and higher poverty rates [1, 2, 4, 5]. Other contributing factors noted in the literature include lifestyle factors, existing chronic conditions, family history, tumor characteristics, a lack of social support, mistrust of medical systems, and perceptions of both racial discrimination and the African-American masculine role [3–14].

In June 2016, the U.S. Preventive Services Task Force (USPSTF), a panel of independent national experts who provide recommendations about clinical preventive services, recommended that all people, unless at heightened risk, should begin obtaining regular CRC screening at age 50 [15, 16]. In May 2018, the American Cancer Society (ACS) recommended that, due to increasing rates of CRC in younger individuals, routine screening should begin at age 45 [17]. Evidence-based screening for CRC exists in the form of either stool-based laboratory tests or camera-aided visual exams of the colon and rectum [17]. These options for asymptomatic individuals who are at average risk include the fecal immunochemical test (FIT) and guaiac-based fecal occult blood test (FOBT), recommended yearly; the multi-targeted stool DNA test every 3 years; a computed-tomography colonography or flexible sigmoidoscopy every 5 years, or a colonoscopy every 10 years [17]. An estimated 50% reduction in CRC mortality among the total U.S. population has been attributed to adherence to these guidelines [18].

Although CRC screening rates have been improving since 2005, evidence suggests that uptake remains low among African-American men and that screening is poorly understood in this population [19, 20]. African Americans “in general” have lower screening rates than whites (55.5% versus 61.5%) [1]. Recent research has identified several key factors associated with lower CRC screening uptake in African-American men, including fear and anxiety, especially in regard to colonoscopy, and a lack of knowledge about the curability of early-stage CRC [13, 21]. African-American men may also overestimate the risks associated with CRC screening procedures [13]. In addition, the FOBT—that requires patients to avoid eating certain kinds of meat and vegetables before the collection of stool, has a lengthy testing time, and necessitates patients handling their own fecal matter—has been associated with negative attitudes among African Americans [22].

Understanding and implementing evidence-based interventions that increase screening uptake among African-American men is a challenge. Intervention studies have reported mixed results about determining the most efficacious methods of increasing CRC screening uptake overall, and few studies of this nature specific to African-American men have been conducted. Furthermore, many of these trials are of low quality. Gee, Walsemann, and Brondolo argue that such interventions should be grounded in a theoretical approach that includes cultural and social factors as agents of change, as all too often healthcare interventions neglect the importance of these aspects in patients’ healthcare decisions [23]. Accordingly, our research team advocated for an assessment and evaluation of current evidence-based interventions that target increasing CRC screening uptake in African-American men. To meet this goal, we conducted a systematic review of the literature and a quantitative meta-analysis, with 2 aims: (1) synthesize the evidence from published studies evaluating interventions to increase CRC screening uptake among African-American men; and (2) quantitatively assess the evidence from these published studies through meta-analysis to determine the most effective screening uptake interventions for African-American men.

### Rationale for systematic literature reviews

In recent decades, the amount of research on interventions to increase the uptake of CRC screening among African-American men has increased exponentially [24]. Consequently, it is more difficult for some researchers and medical professionals to digest current findings and directions in the literature [24]. Systematic reviews thus fill an important role by providing an overview of the current state of research on a particular topic, pointing out weaknesses and gaps in the literature, and clarifying where disagreement or contradictions are reported, as findings from individual studies may be inconsistent [25, 26]. Systematic methods must be used to conduct literature reviews because nonsystematic or narrative reviews are difficult to properly assess [27]. The use of a systematic approach also permits the researcher to create a set of parameters that allows for the elimination of bias by excluding flawed studies from the ultimate analysis [27].

### Rationale for meta-analyses

Single studies are informative for building the literature on a particular topic, especially when evaluating treatments and interventions, but are prone to false positives and negatives [25]. A meta-analysis provides a systematic approach for evaluating a series of studies in which those with larger sample sizes carry more weight [25]. The strength of this technique also includes 1) enabling researchers to provide a quantitative estimate for the effect of a treatment or intervention, 2) helping researchers identify more precise estimates of intervention effectiveness or other outcomes than any individual study in a pooled analysis [28]. Thus, by aggregating the findings of many studies and correcting for potential error or bias, a meta-analysis helps to identify the potential pitfalls within any single study as well as to identify studies with consistent results [24]. Additionally, this technique enables researchers to identify the overall effect of a particular treatment or intervention [25].

## Materials and methods

### Study selection

To be included in the review, studies had to be: (1) inclusive of African-American/Black adult men 18 years of age or older; (2) focused on interventions for CRC screening uptake; (3) published after 1998, 2 years before the initial publication of the American College of Gastroenterology’s CRC screening guidelines [29]; (4) written in English and conducted in the U.S.; and (5) published, peer-reviewed, full-text journal articles. The (colorectal) cancer profile of African-born Blacks differs from that of U.S. States-born Blacks and also varies by region of birth [30], thus, our research team solely focused on U.S.-born African-American men. Studies based on secondary analyses of data were excluded. Although potential for publication bias remains, only peer-reviewed articles were considered as review for quality, relevancy, and accuracy by multiple experts in the field provides a higher level of validity [31]. In addition, because the research team was interested in interventions that proved to be effective in achieving increased screening completion, included studies had to report on actual CRC screening uptake, as opposed to changes in CRC screening beliefs or intent to screen.

To determine an article’s eligibility, a team consisting of 3 systematic review screening-trained co-investigators (authors CR, MH, and PM) performed 2 rounds of assessment. In the first round, they reviewed potentially eligible studies by article title and abstract from the results obtained in Rayyan QCRI, a web-based systematic-review platform that was created to expedite the initial screening of abstracts and titles, and uses a process of semi-automation while incorporating a high level of usability [32]. The databases Medline (Ovid), CINAHL (EBSCO), Embase (Ovid), and Cochrane CENTRAL (Wiley) were individually searched, and Rayyan QCRI was used to sort the retrieved manuscripts.

Last ran on January 14, 2020, the literature search combined 4 concepts: colorectal cancer, screening, African American, and men. Each concept was searched using database thesaurus terms and keywords as appropriate (see S1 Appendix). This method was based on the principles discussed in the Cochrane Handbook, including the combination of keywords and subject headings [33]. Discrepancies between the 2 team members were adjudicated by the study Principal Investigator (PI; first author CRR).

After this initial screening process, articles that potentially met all criteria, or the eligibility of which was unclear, underwent a second round of screening in which the same 2 authors again independently screened each article, and conflicts were resolved through face-to-face meetings with the PI. The screening team also reviewed the references of included articles. As a systematic review, this study did not require informed consent or institutional review board approval as human subjects were not involved. Yet, the protocol was registered with the International Prospective Registry of Systematic Reviews (PROSPERO 2019 CRD42019119510).

### Data abstraction, risk of bias assessment, synthesis, and analysis

After articles had been accepted for the systematic review, the same 3 members of the research team (CR, MH, PM) coded each paper individually by entering information into a standardized Google form. Extracted data covered study characteristics such as sample size, demographics, and eligibility criteria. Other data gathered from the accepted articles included statistical analyses used, intervention type, theoretical background used, and limitations cited by the study authors. CR, MH, and PM also abstracted data and met weekly to resolve any coding conflicts. Tables were constructed to qualitatively describe the study design and report the results of each included study.

In order to determine the potential risk of bias, each study was randomly assigned to 2 of 3 study members (CRR, PM, MF) who assessed the studies by applying the appropriate critical appraisal criteria based on each study’s type independently. During this quality assessment process, the two authors followed the blind review protocol [34]. In detail, Cochrane Collaboration’s checklist was used to assess risk of bias for randomized control trials [35], as well as Joanna Brigg’s checklist for quasi-experimental [36] and cohort [37] studies. All disagreements were settled through discussions with a third member until consensus was reached.

For the meta-analysis, we first defined the values of I^2^ statistics; our results showed that I^2^ >75%, indicating that considerable heterogeneity was present [38, 39]. Next, substantial heterogeneity was investigated using meta-regression for intervention type (control intervention, FIT or other stool-based screening test, printed education materials, or patient navigation) as recommended by Sharp [40] and Newton [41]. Control intervention denoted the control group in the studies. For example, some studies reported usual care as a control group while others mailed letters recommending CRC screening. Then, we conducted sensitivity analyses to evaluate the effect of removing any 1 study from each meta-regression. To evaluate for bias resulting from the absence of studies with negative or insignificant results (often termed “publication bias”), we examined funnel plots visually for asymmetry and used the method of Egger and colleagues to formally test for funnel-plot asymmetry [25]. The “metaprop” and "metareg” functions in R package “meta” and “metafor” were employed for statistical analysis, using a random intercept logistic regression model that was fitted using restricted maximum likelihood (REML). We also used Clopper-Pearson exact binomial confidence intervals to report, respectively, for individual studies on the funnel plots. These analyses were conducted using R, and figures were produced using the meta package [42–44].

## Results

### Sample

**Fig 1:**
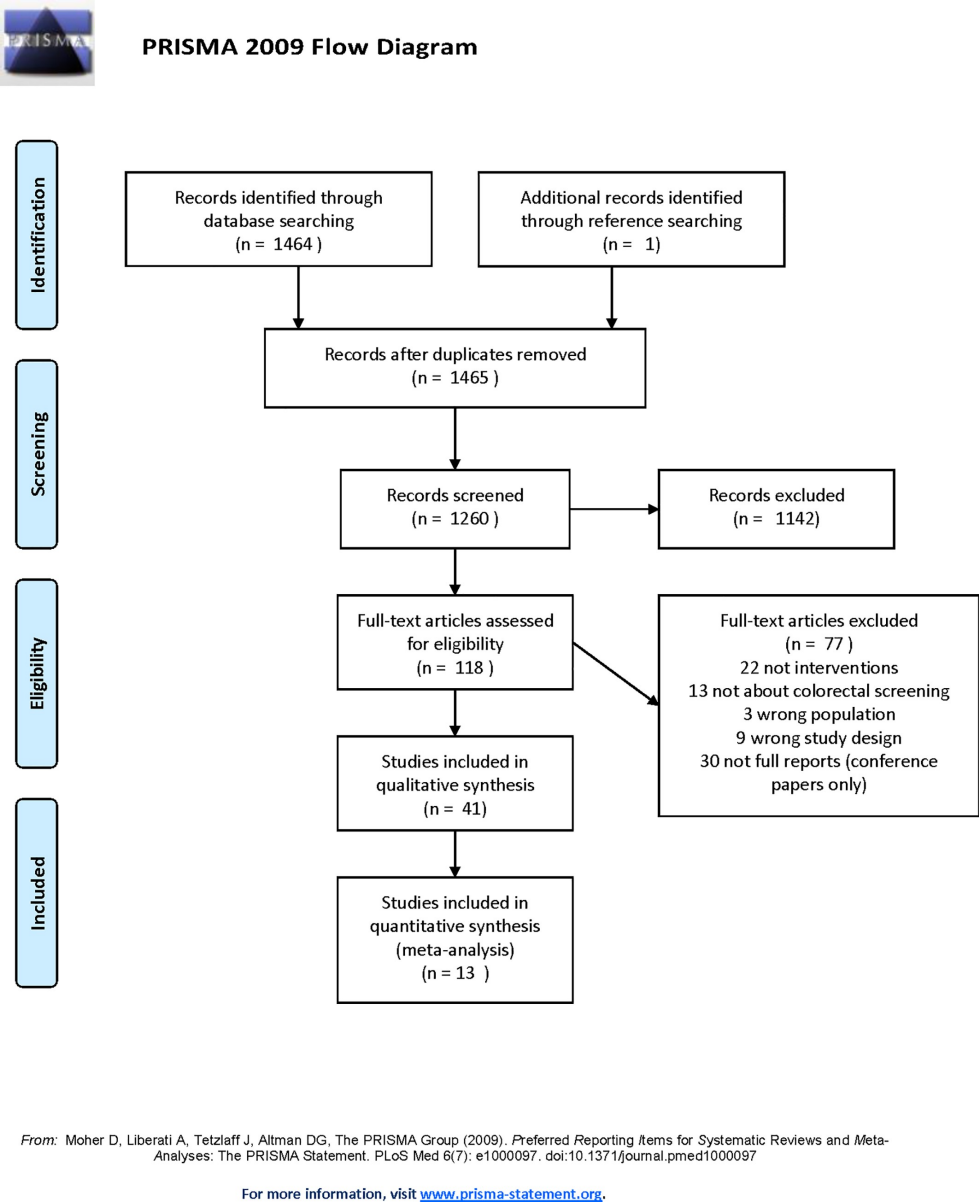
Selection of studies for inclusion in review and meta-analysis.

A total of 1,465 articles were initially identified from the 4 databases searched, 41 (2.79%) were included in the final sample for the systematic review [45–85]. As recommended by the PRISMA group [86], Fig 1 provides details regarding the identification, screening, eligibility, and inclusion processes. S2 Appendix details the research team’s adherence to PRISMA’s checklist—which helped improve the reporting quality for the current study.

### Characteristics of included studies

Most interventions (73%) were performed in clinical or medical facilities. The remaining 11 (27%) studies that were not conducted in a clinical setting took place in a church (12%), business (3%), or did not report their intervention setting (12%). Studies were published between 2000 and 2019, with most (n = 12) appearing between 2010 and 2012. Two authors published more than 1 study on the topic (11%), namely, Blumenthal (n = 2), and Leone (n = 2) [50, 54, 68, 77]. Slightly more than half (56%; n = 23) of the studies included only participants aged 50 years and older, while five (12%) included participants starting at age 45 years, three (7%) included participants younger than 45. Twelve of the studies (29.27%) reported demographics specific to African-American men [50, 52, 56, 57, 59, 60, 63, 66, 71, 74, 82, 84]. In the studies aimed at increasing CRC screening uptake among African-American men, the intervention components most frequently employed (among 135 interventions types utilized) were telephone encounters or education (18%; n = 25), mailed or electronically sent educational materials (13%; n = 18), FIT or other CRC stool-based screening kits (mailed or administered in person) (13%; n = 17), patient navigation (10%; n = 13), and printed materials given to individuals in person (12%; n = 16). A matrix of the included studies, sorted according to their theoretical design and methodological features, is found in Table 1; these features are detailed below.

**Table 1:** Matrix of 41 reviewed studies, according to theoretical design and methodological features.

Study	Theoretical Framework	Study Design	Most Advanced Statistical Analysis	Validly and Reliably Reported	Number of African-American Male Participants Reported
Arnold et al. (2019)	Explicit use	RCT	Multivariate evaluation	Validity: No Reliability: No	Not reported
Basch et al. (2006)	No framework	RCT	Descriptive	Validity: No Reliability: No	Not reported
Bastani et al. (2015)	Explicit use	RCT	Multivariate evaluation	Validity: No Reliability: No	Yes
Blumenthal at al. (2005)	Explicit use	Quasi-experimental	Multivariate evaluation	Validity: No Reliability: No	Not reported
Blumenthal et al. (2010)	Explicit use	RCT	Chi-square tests	Validity: No Reliability: No	Yes
Chen et al. (2008)	No framework	Cohort	Chi-square tests	Validity: No Reliability: No	Not reported
Christy et al. (2016)	Explicit use	RCT	Multivariate evaluation	Validity: No Reliability: No	Not reported
Cole et al. (2017)	No framework	RCT	Multivariate evaluation	Validity: No Reliability: No	Yes
Davis S et al. (2017)	Explicit use	RCT	Multivariate evaluation	Validity: No Reliability: No	Not reported
Davis T et al. (2019)	Explicit use	RCT	Multivariate evaluation	Validity: No Reliability: No	Not reported
DeGroff et al. (2017)	Explicit use	RCT	Multivariate evaluation	Validity: Yes Reliability: No	Not reported
Eberth et al. (2018)	No framework	Cohort	Descriptive	Validity: No Reliability: No	Not reported
Fiscella et al. (2011)	No framework	RCT	Multivariate evaluation	Validity: No Reliability: No	Not reported
Ford et al. (2006)	Explicit use	RCT	Chi-square tests	Validity: No Reliability: No	Yes
Greiner et al. (2014)	Explicit use	RCT	Multivariate evaluation	Validity: No Reliability: No	Not reported
Gupta et al. (2013)	No framework	RCT	Chi-square tests	Validity: No Reliability: No	Not reported
Hendren et al. (2014)	Explicit use	RCT	Multivariate evaluation	Validity: No Reliability: No	Not reported
Hoffman et al. (2017)	Explicit use	RCT	Multivariate evaluation	Validity: No Reliability: No	Yes
Holt et al. (2011)	Explicit use	Quasi-experimental	Multivariate evaluation	Validity: No Reliability: No	Not reported
Horne et al. (2015)	No framework	RCT	Multivariate evaluation	Validity: No Reliability: No	Yes
Inadomi et al. (2012)	No framework	RCT	Multivariate evaluation	Validity: No Reliability: No	Not reported
Jandorf et al. (2013)	Explicit use	RCT	Multivariate evaluation	Validity: No Reliability: No	Yes
Kempe et al. (2012)	No framework	Quasi-experimental	Multivariate evaluation	Validity: No Reliability: No	Not reported
Khankari et al. (2007)	No framework	Quasi-experimental	Chi-square tests	Validity: No Reliability: No	Not reported
Lasser et al. (2011)	Explicit use	RCT	Chi-square tests	Validity: No Reliability: No	Not reported
Leone et al. (2013)	No framework	Quasi-experimental	Multivariate evaluation	Validity: No Reliability: No	Not reported
Leone et al. (2016)	Explicit use	RCT	Multivariate evaluation	Validity: No Reliability: No	Not reported
Martin et al. (2017)	Explicit use	Quasi-experimental	Descriptive	Validity: No Reliability: No	Not reported
Maxwell et al. (2019)	No framework	RCT	Descriptive	Validity: No Reliability: No	Yes
Mehta et al. (2019)	Explicit use	Quasi-experimental	Descriptive	Validity: No Reliability: No	Not reported
Miller et al. (2011)	Explicit use	RCT	Multivariate evaluation	Validity: No Reliability: No	Not reported
Morgan et al. (2010)	Explicit use	RCT	Multivariate evaluation	Validity: No Reliability: No	Yes
Myers et al. (2014)	Explicit use	RCT	Multivariate evaluation	Validity: No Reliability: No	Yes
Philip et al. (2010)	Explicit use	Quasi-experimental	Descriptive	Validity: No Reliability: No	Not reported
Pignone et al. (2011)	No framework	RCT	Multivariate evaluation	Validity: No Reliability: No	Not reported
Resnicow et al. (2014)	Explicit use	RCT	Multivariate evaluation	Validity: No Reliability: No	Yes
Reuland et al. (2017)	No framework	RCT	Multivariate evaluation	Validity: No Reliability: No	Not reported
Schroy et al. (2012)	Explicit use	RCT	Multivariate evaluation	Validity: No Reliability: No	Not reported
Siddiqui et al. (2011)	Explicit use	RCT	Multivariate evaluation	Validity: No Reliability: No	Yes
Singal et al. (2017)	No framework	RCT	Chi-square tests	Validity: No Reliability: No	Not reported
Zubarik et al. (2000)	No framework	Cohort	Descriptive	Validity: No Reliability: No	Not reported

#### Theory

Nearly half of the studies (39%; n = 16) did not report a theoretical framework, while the remaining studies (61%; n = 25) used 1 to 3 frameworks. A theoretical foundation built on 1 conceptual model was most common (56%; n = 14), with the Health Belief Model the most utilized (28%; n = 7), followed by the Preventive Health Model (16%; n = 4) and the Transtheoretical Model (also referred to as Stages of Change) (16%; n = 4). For example, in a study grounded by the Preventive Health Model and input from a community advisory board, Christy and colleagues tested the effectiveness of their self-created CRC photonovella booklet plus a fecal immunochemical test (FIT) kit and the CDC’s standard “Screen for Life” brochure (not targeted to African-Americans plus a FIT kit among 330 Black participants (308 African Americans, 22 Caribbean/Haitians/Other) in Florida who were not current with CRC screening [5]. Fifty-two percent of this sample was male. The educational messages developed for the CRC photonovella were based on the following theoretical constructs: barriers, self-efficacy, CRC screening coherence and salience, response efficacy, and susceptibility of focus. Also, noteworthy, a randomized control trial (RCT) by DeGroff and colleagues was the only study driven by 3 frameworks: the Health Belief Model, Theory of Reasoned Action, and social learning theories [73]. This patient-navigation intervention aimed to address multilevel patient-defined barriers to CRC screening completion among 840 patients who were referred for a colonoscopy by primary care providers in Massachusetts. Eighty percent of participants were either non-Hispanic Black (40%) or Hispanic (40%). Two bilingual lay navigators (1 male, 1 female) delivered the intervention primarily via telephone, while some activities were conducted by mail or in person, with an average time of 44 minutes per patient. Both navigators received additional training in motivational interviewing.

#### Geography

Interventions were evaluated by geographic location, with 4 regions considered during coding: West, Midwest, South, and Northeast [87]. Interventions were conducted in all 4 geographic regions: South (44%), Northeast (32%), Midwest (12%), and West (12%). Most studies (90%; n = 37) reported that intervention delivery occurred in a single geographic region, 2 articles reported delivery in 2 regions, and 2 articles did not report the location of their interventions. For example, Leone and colleagues performed their intervention in both Michigan and North Carolina, stating that the 2-state approach was as an effort to increase the generalizability of their results [54].

#### Setting

Setting—where studies take place—is a highly influential factor for intervention outcomes due to its potential impact on sample representation, intervention uptake, and sustainability. Approximately 81% of studies implemented interventions in 1 primary setting, 7% used multiple settings, and 12% did not report the intervention setting. For example, Blumenthal and colleagues delivered CRC education messages via radio, newsletters, public transportation, health fairs, festivals, television programs, etc [68]. Blumenthal and colleagues used community-based participatory research, which places the responsibility for intervention design and delivery, including setting, in the hands of community partners [50, 68]. DeGroff et al., Fiscella and colleagues, and Gupta et al. are examples of studies that used local resources (i.e. established medical networks and safety clinics) to meet the disproportionate health needs of largely minority areas [46, 58, 73]. Two studies used clinical/medical settings due to the presence of an open-access endoscopy system, which was defined by Chen and colleagues as providing direct referrals for CRC screening and thus bypassing additional gastroenterology exams and decreasing the number of appointments that study participants needed to attend [47, 49].

Interventions that took place outside of clinical or medical settings were conducted in churches (12%), local businesses (5%), and other communal spaces (e.g., community centers, social organizations) (~5%). Several studies justified using church-based settings because religion and faith are central themes of African-American culture; church settings are a natural venue to introduce faith-based intervention materials; previous church-based interventions have proven effective for addressing other health disparities in African-American populations; and church settings may be able to reach those with limited access to healthcare [54, 60, 78]. Cole and colleagues, who conducted their intervention in New York City barbershops, emphasized the community-based approach, stating that––as is commonly seen among African-American men––those with the greatest need are not accessing the healthcare system [57]. Setting selection in our sample was likely also influenced by available resources, funding sources, and population need. The effectiveness of interventions conducted in clinical versus community-based settings should be further evaluated.

#### Post-intervention screening uptake

Reported screening uptake percentages among study participants ranged from 8% to 90% [45, 76]. The intervention with the highest overall screening uptake (81.9%) involved sending participants a culturally sensitive photonovella and a free FIT kit [45]. Interestingly, however, this same study reported that 90% of their controls––subjects who received a standard CRC screening brochure developed by the CDC, along with the free FIT kit––also reported screening uptake [45]. Most, but not all, studies reported higher screening uptake rates in the intervention group than in the control group.

#### Intervention types

Many of the studies evaluated used more than 1 intervention to increase CRC screening (see Table 2). Often, 2 or more intervention arms were used, along with a control arm. Intervention arms often included more than 1 component or type of intervention. For example, 1 study compared patient navigation alone with patient navigation plus motivational interviewing or a control group [57]. Another study combined a screening decision aid with patient navigation [62]. One intervention was a citywide messaging campaign that included educational sessions at local community centers, yard signs, and messages in newspapers [68]. Because of this heterogeneity, it was difficult to succinctly categorize studies by the type of intervention used. However, we found that 43% (n = 16) of studies used some form of tailored educational material; 32% (n = 12) included patient navigation; 22% (n = 9) included some form of group or 1-on-1 education; 22% (n = 9) gave subjects free FIT or immunochemical fecal occult blood test (iFOBT) kits; 9% (n = 4) employed telephone outreach; and 3% (n = 1) intervened at the physician level.

**Table 2:** Number of CRC interventions used in a sample of (41) reviewed studies.

Intervention Type (n = # of studies)	Study			
Telephone encounter/ education (n = 25)	Arnold et al (2019)	Eberth et al. (2018)	Lasser et al. (2011)	Reuland et al. (2017)
	Basch et al. (2006)	Fiscella et al. (2011)	Leone et al. (2013)	Siddiqui et al. (2011)
	Bastani et al. (2015)	Ford et al. (2006)	Leone et al. (2016)	Singal et al. (2017)
	Chen et al. (2008)	Gupta et al. (2013)	Martin et al. (2017)	Zubarik et al. (2000)
	Christy et al. (2016)	Hendren et al. (2014)	Maxwell et al (2019)	
	Cole et al. (2017)	Jandorf et al. (2013)	Mehta et al (2019)	
	DeGroff et al. (2017)	Kempe et al. (2012)	Myers et al. (2014)	
Mailed/electronic version of education materials (n = 18)	Christy et al. (2016)	Hendren et al. (2014)	Leone et al. (2016)	
	Cole et al. (2017)	Jandorf et al. (2013)	Pignone et al. (2011)	
	Davis T. et al (2019)	Kempe et al. (2012)	Resnicow et al. (2014)	
	DeGroff et al. (2017)	Lasser et al. (2011)	Siddiqui et al. (2011)	
	Fiscella et al. (2011)	Leone et al. (2013)	Singal et al. (2017)	
	Gupta et al. (2013)	Myers et al. (2014)	Zubarik et al. (2000)	
FIT/iFOBT/Stool test kit (n = 17)	Christy et al. (2016)	Gupta et al. (2013)	Leone et al. (2016)	Siddiqui et al. (2011)
	Cole et al. (2017)	Hendren et al. (2014)	Martin et al. (2017)	Singal et al. (2017)
	Davis T. et al (2019)	Inadomi et al. (2012)	Mehta et al. (2019)	
	Fiscella et al. (2011)	Kempe et al. (2012)	Myers et al. (2014)	
	Greiner et al. (2014)	Lasser et al. (2011)	Reuland et al. (2017)	
Patient Navigation (n = 13)	Chen et al. (2008)	Fiscella et al. (2011)	Lasser et al. (2011)	Reuland et al. (2017)
	Cole et al. (2017)	Ford et al. (2006)	Leone et al. (2013)	
	DeGroff et al. (2017)	Horne et al. (2015)	Martin et al. (2017)	
	Eberth et al. (2018)	Jandorf et al. (2013)	Myers et al. (2014)	
Printed Materials (n = 16)	Arnold et al. (2019)	Christy et al. (2016)	Inadomi et al. (2012)	Miller et al. (2011)
	Bastani et al. (2015)	Davis S et al (2017)	Khankari et al. (2007)	Morgan et al. (2010)
	Blumenthal at al. (2005)	Davis T et al (2019)	Leone et al. (2016)	Philip et al. (2010)
	Blumenthal et al. (2010)	Holt et al. (2011)	Maxwell et al. (2019)	Reuland et al. (2017)
One-on-One Education (n = 13)	Arnold et al. (2019)	Fiscella et al. (2011)	Inadomi et al. (2012)	Reuland et al. (2017)
	Blumenthal et al. (2010)	Hendren et al. (2014)	Maxwell et al. (2019)	
	Davis S et al. (2017)	Holt et al. (2011)	Martin et al. (2017)	
	Eberth et al. (2018)	Khankari et al. (2007)	Philip et al. (2010)	
Decision Aid (n = 6)	Hoffman et al. (2017)	Miller et al. (2011)	Reuland et al. (2017)	
	Leone et al. (2013)	Pignone et al. (2011)	Schroy et al. (2012)	
Group Education (n = 5)	Blumenthal at al. (2005)	Holt et al. (2011)	Morgan et al. (2010)	
	Blumenthal et al. (2010)	Leone et al. (2016)		
Community Outreach (n = 4)	Blumenthal at al. (2005)	Leone et al. (2016)	Martin et al. (2017)	Morgan et al. (2010)
DVD/Video (n = 4)	Hoffman et al. (2017)	Leone et al. (2016)	Martin et al. (2017)	Morgan et al. (2010)
Educate/Train Clinical Staff (n = 4)	Martin et al. (2017)	Pignone et al. (2011)	Schroy et al. (2012)	Zubarik et al. (2000)
Case Management (n = 1)	Ford et al. (2006)			
Financial Assistance (n = 1)	Blumenthal et al. (2010)			
Financial Incentive (n = 1)	Mehta et al. (2019)			
Interactive Kiosk (n = 1)	Greiner et al. (2014)			
Open Access Colonoscopy (n = 1)	Eberth et al. (2018)			
Peer Counselors (n = 2)	Leone et al. (2016)	Maxwell et al (2019)		
Resource Sheet (n = 1)	Leone et al. (2016)			
Online Risk Assessment (n = 1)	Schroy et al. (2012)			
Sigmoidoscopy Program (n = 1)	Zubarik et al. (2000)			

The intervention that performed best compared with controls was in a study by Reuland and colleagues that incorporated a screening decision aid along with patient navigation; 68% of participants in the intervention arm reported screening uptake compared with 27% of controls [62]. Unfortunately, this study did not break out its screening-uptake results by race; thus, it cannot be determined how much this intervention affected African Americans specifically. Among African Americans specifically, the intervention that resulted in the highest screening uptake compared with controls mailed a free FIT kit to participants, 43% of whom returned the kit, compared with 15% of controls who engaged in some other form of screening [58].

The 2 interventions that most commonly reported significant differences in screening uptake among African-American participants compared with controls were patient navigation and free FIT or iFOBT kits. Tailored materials, such as culturally sensitive brochures or videos, and education, whether in person or in a group setting, also had positive outcomes, but not to the extent of patient navigation or free screening kits. Interestingly, Mehta and colleagues approached CRC screening uptake by providing financial incentives via 3 arms: unconditional, conditional, and lottery [81]. The unconditional arm was given a $10 gift card along with the FIT kit, while those in the conditional arm received the $10 gift card after completion of the FIT testing if within 2 months. Participants randomized to the lottery arm were afforded the opportunity to win a $100 gift card–with a 1 in 10 chance of winning—if the FIT test were completed and returned within the 2 months mark [81].

#### Intervention delivery

Interventions were most commonly implemented by the study researchers themselves, medical personnel, or public health workers (as was the case among patient navigation interventions). Some studies enlisted the help of local community members, including churches, barbershops, and other local businesses [57, 68, 78, 82]. Besides the use of patient navigators in general, there was no observed trend in CRC screening uptake related to who implemented the intervention. For example, standard patient navigation resulted in higher CRC screening uptake (80%) compared with peer–patient navigators (African-American community members who were specially trained to be patient navigators) (74%) or pro–patient navigators (healthcare professionals who were trained to deliver culturally sensitive patient navigation) (76%) [60].

#### Limitations of interventions

Most studies recognized a lack of generalizability in their results due to the subject population or the geographic region in which the study was conducted. For example, 1 study reported that “participants were recruited from healthcare providers in large urban settings, consisted of 75% females and had regular contact with a healthcare provider, and thus are not representative of the African American community in its entirety [67].” Another reported issue regarding generalizability relates to the potential for selection bias, especially in studies in which the main intervention was a free FIT/iFOBT kit. These studies first contacted the individuals to see if they were willing to participate in the study, and then the subjects received the free kits. Because the participants were already willing to participate in research, they may have been more likely than other members of the general public to use and send back the FIT kits.

In many studies, especially those that used patient navigation or provided free FIT/iFOBT kits, cost was often cited as problematic for wide-scale implementation of the intervention. A study of 2 citywide interventions that implemented various intervention strategies reported that radio and TV were the most effective media used, but recognized these are expensive options that may not be financially feasible for others [68]. However, if granted financial incentives, Mehta et al. found the incentive amount of $10 might have been too small to promote any increase in FIT kit completion [81].

Another commonly reported limitation was the nature of the intervention follow-up. In general, follow-up to check CRC screening status ranged from 3 to 12 months. However, it is possible that participants obtained screening after these follow-up points and thus their data were missed. Along with follow-up time, the ability to contact participants at follow-up was also reported to be problematic. This was especially true in low-income populations. In 1 study, patient navigators were unable to follow up with 23% of participants [55]. This problem was avoided in studies that had access to electronic health records where screening status could easily be verified. However, not all studies had such capability.

Study design was also commonly mentioned as a potential limitation. Though most studies were RCTs, few incorporated any kind of blinding because of the nature of the interventions used (patient navigation, free FIT kits, culturally tailored brochures).

### Risk of bias

**Fig 2:**
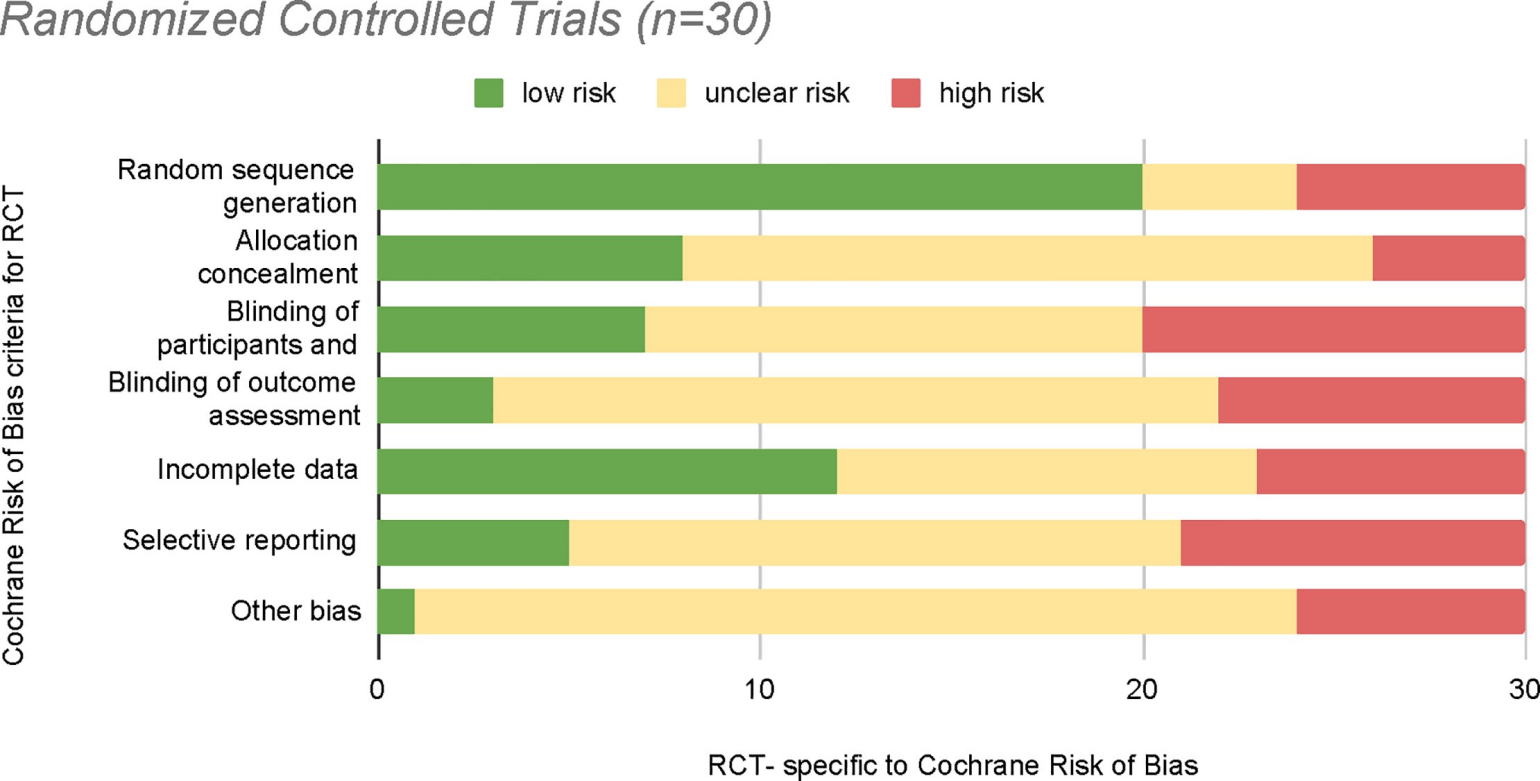
Risk of bias graph for RCTs.

**Fig 3:**
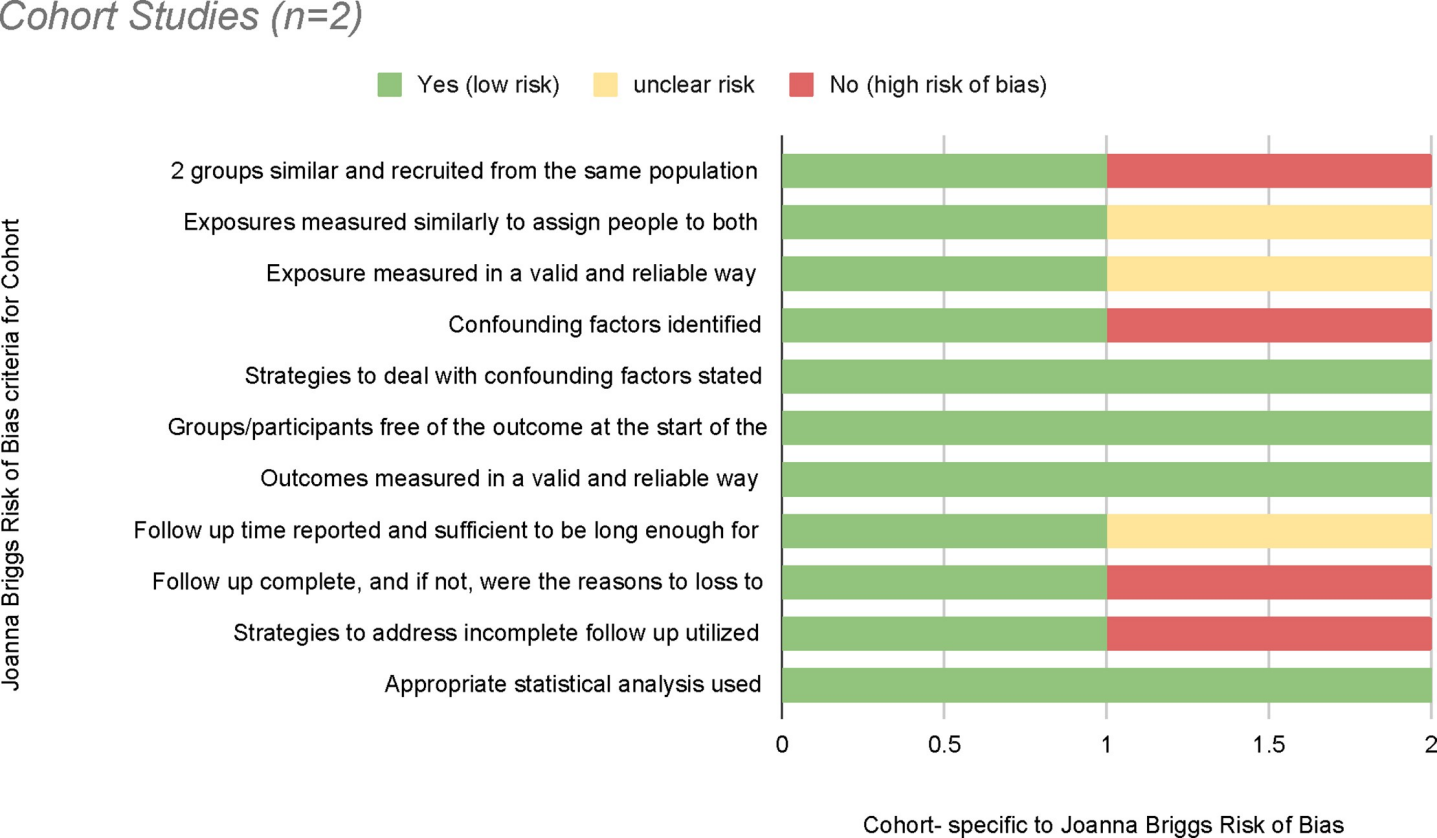
Risk of bias graph for cohort studies.

**Fig 4:**
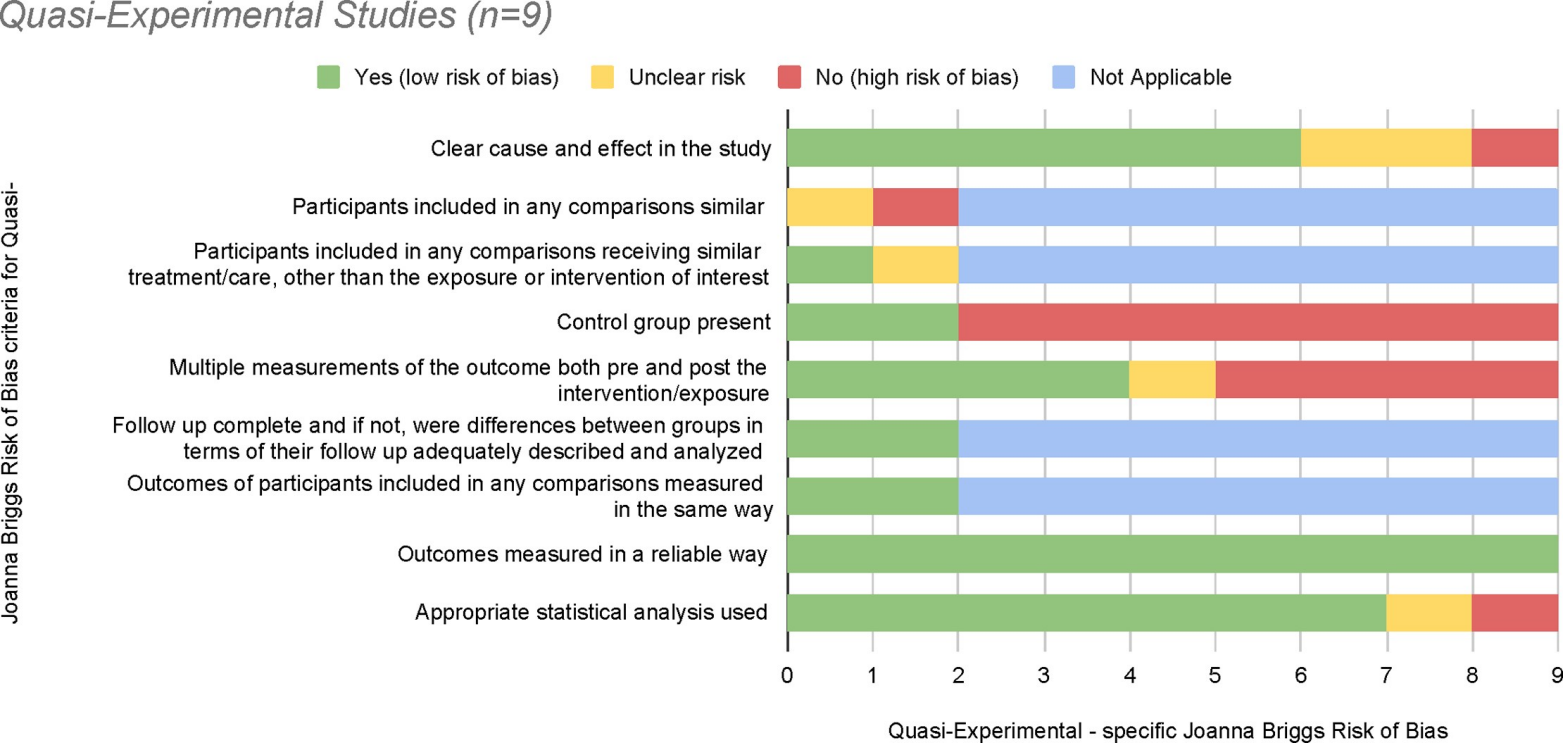
Risk of bias graph for Quasi-experimental studies.

Studies were allocated into 3 categories to assess for biases: Randomized Controlled Trials ([RCTs], Fig 2), Cohort (Fig 3), and Quasi-Experimental (Fig 4). Twenty-nine studies [45, 46, 48, 50–52, 54–66, 69, 71, 73–75, 77, 79–81, 83, 84] in the RCT category were largely rated as unclear risk, yet 20 of the studies had low risk on random sequence generation (i.e., description of randomization procedure) [50–52, 56–63, 69, 74, 75, 79–81]. The majority of the studies (*n* = 13) did not explain or detail if any blinding of the participants and/or personnel in group allocation occurred, which included 18 studies with an unclear risk on allocation concealment [48, 50, 52, 54, 55, 57, 63–66, 71, 74, 77, 79, 80, 83]. Either listed as a supplement in the manuscript or published elsewhere, only 5 studies [50, 57, 58, 61, 80] reported their study protocol. Overall, 26 RCTs did not account for blinding of outcomes while 6 RCTs [45, 48, 50, 56, 57, 63] received a high bias rating due to their report of additional biases. Moreover, studies with the highest numbers of interventions (*n* = 9, Leone et al. 2016 [54], *n* = 7, Martin et al. 2017 [72], and *n* = 6, Reuland et al. 2017 [62]) demonstrated risk of bias scores ranging from unclear to high risks. Multicomponent studies with the lowest risk bias utilized 4 or fewer types of interventions.

Next, 9 of the studies were classified as quasi-experimental [49, 53, 67, 68, 70, 72, 76, 78, 82]. Although only 2 of these studies contained a control group [68, 76], a high bias risk was yielded for the remaining 7 studies. All 9 studies revealed low bias risk for outcome measurement appropriate statistical analysis use. Lastly, 2 studies were considered for the cohort design [47, 85], with both having similar ratings for low bias risk.

### Meta-analyses

**Fig 5:**
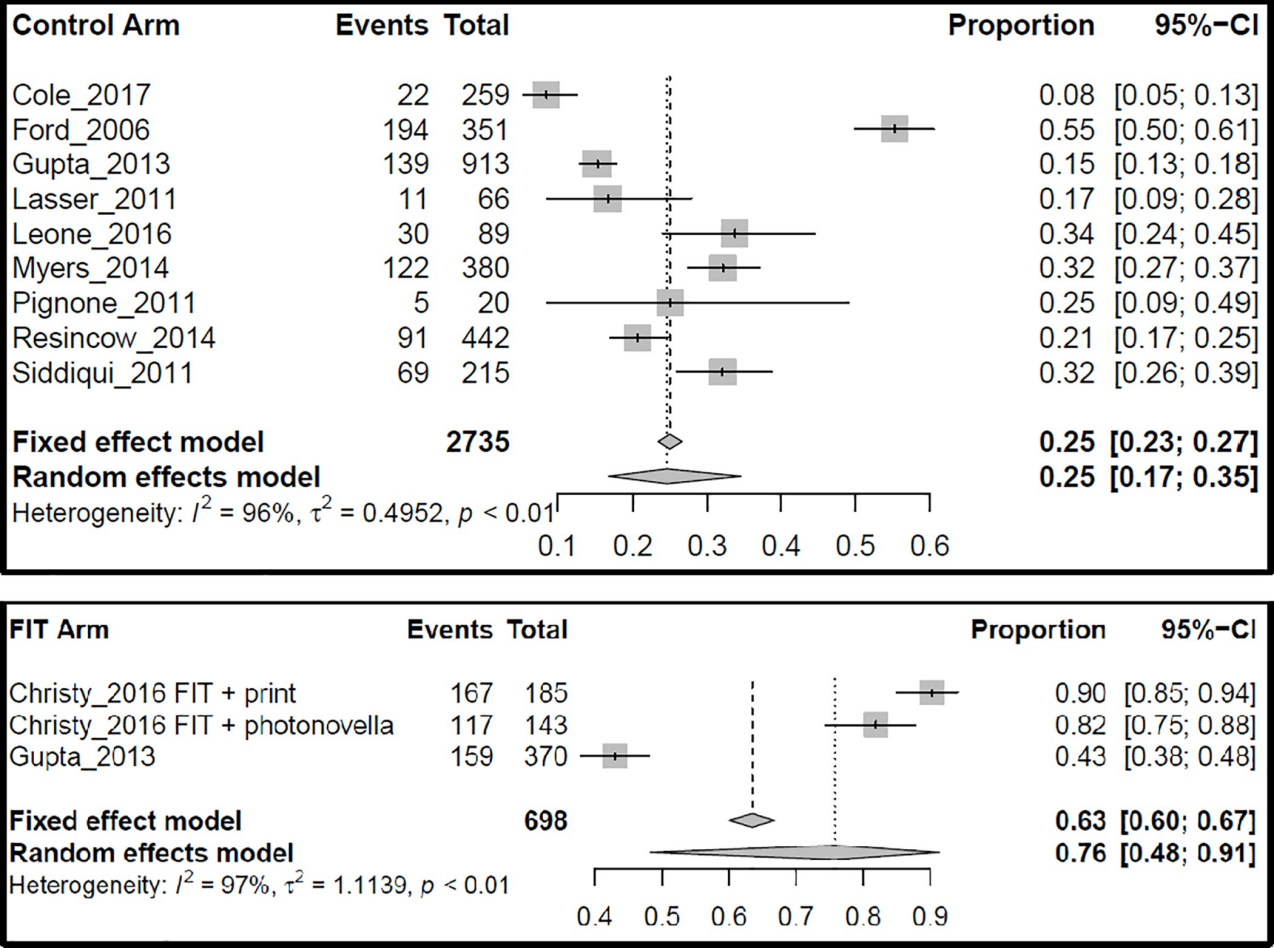
Meta-analysis for control and FIT (fecal immunochemical test) interventions. **Events**: number of participants with screening outcomes in the arm of the study; **Total**: number of participants in the arm of the study; **Proportion**: the ratio between Events and Total.

**Fig 6:**
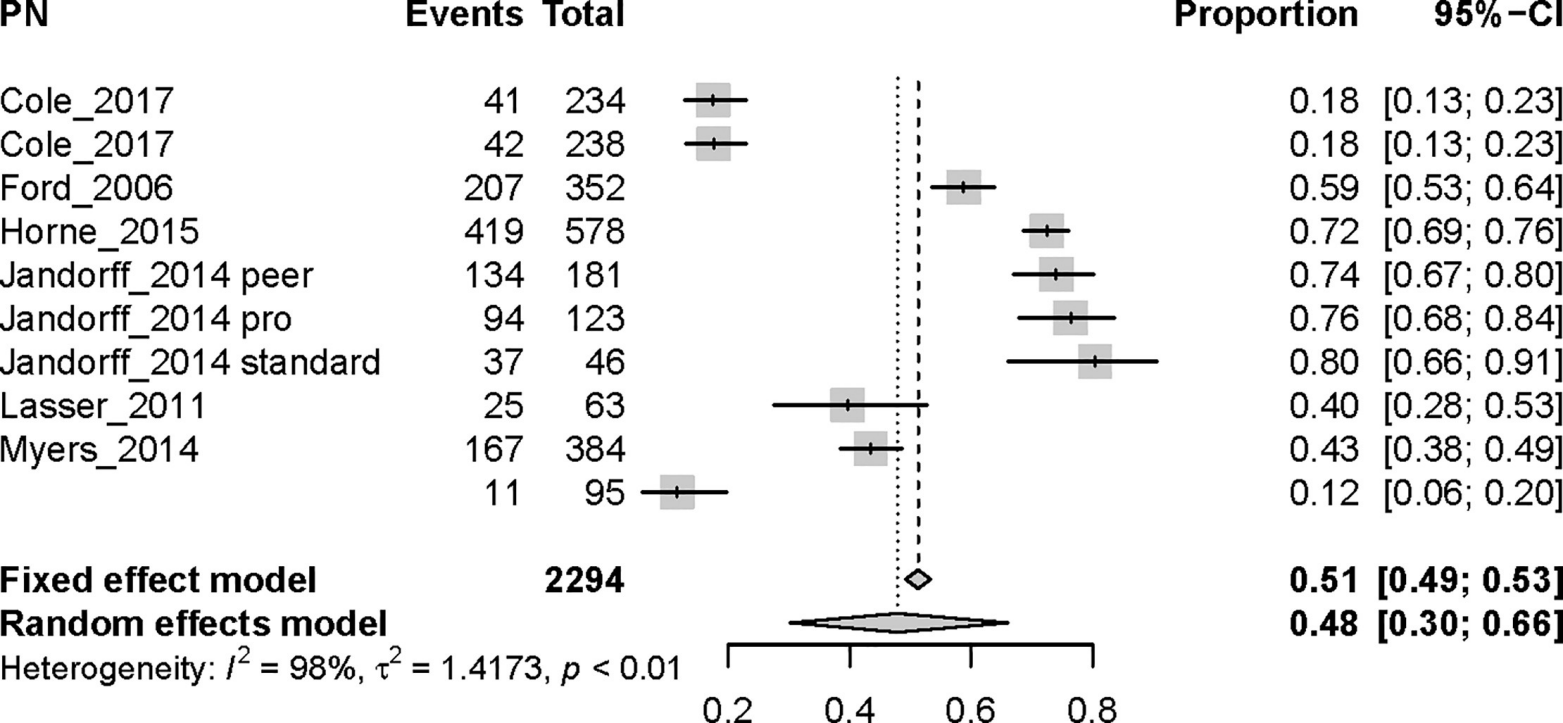
Meta-analysis for PN (patient navigation) interventions. **Events**: number of participants with screening outcomes in the arm of the study; **Total**: number of participants in the arm of the study; **Proportion**: the ratio between Events and Total.

**Fig 7:**
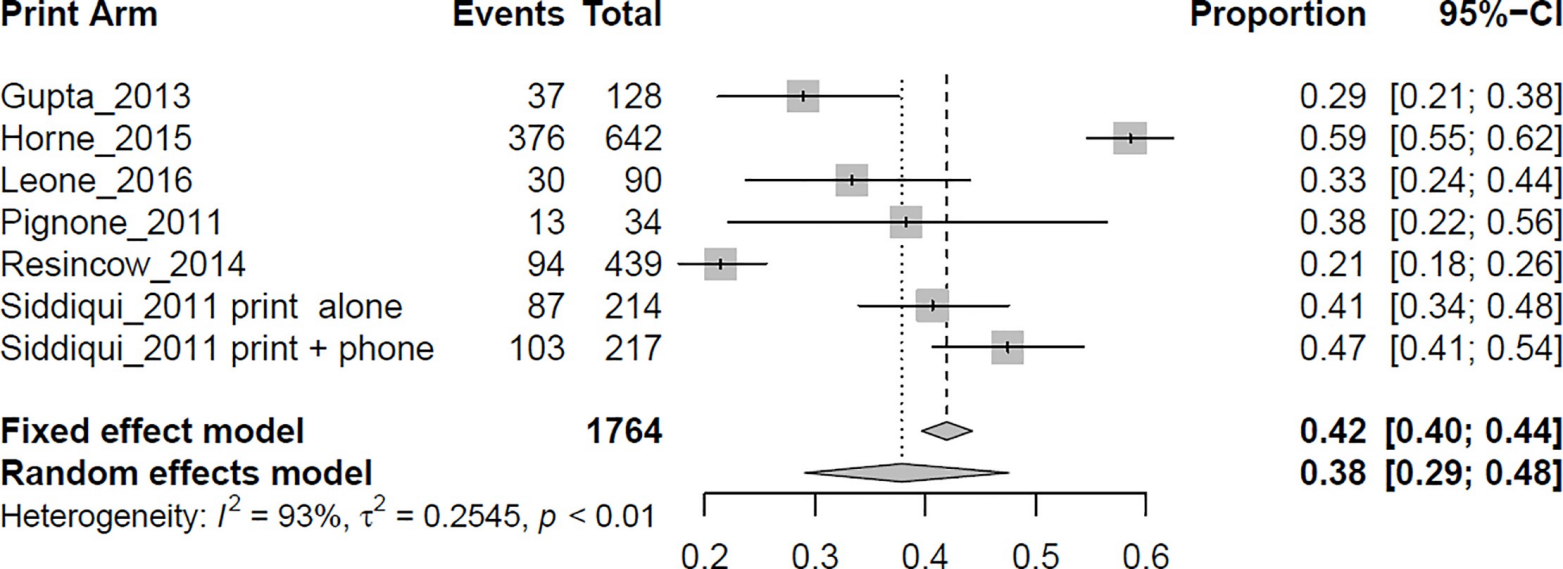
Meta-analysis for print interventions. **Events**: number of participants with screening outcomes in the arm of the study; **Total**: number of participants in the arm of the study; **Proportion**: the ratio between Events and Total.

Meta-regression was used to compare the 4 intervention types (Table 3). The endpoint for the meta-analysis was intervention effectiveness, defined as the proportion of participants that obtained CRC screening during or after the intervention. Interventions that used a FIT kit or patient navigation were significantly better than the print and control intervention at increasing CRC screening uptake among African-American men, with odds ratios (ORs) of 9.60 (95% CI 2.89–31.82, p = 0.0002) and 2.84 (95% CI 1.23–6.49, p = 0.01), respectively. Meta-analysis results were reported for each intervention type separately (see Figs 5–7). Interventions that used print materials were not significantly better than control interventions. To directly compare pairs of interventions, additional models were fitted restricting the studies to a pair of interventions. In these models, FIT interventions were not superior to patient-navigation (PN) interventions (OR = 3.42, 95% CI 0.75–15.62, p = 0.11), FIT interventions were superior to print interventions (OR = 5.01, 95% CI 1.87–13.67, p = 0.001), and PN interventions were not significantly different from print interventions (OR = 1.52, 95% CI 0.59–3.90, p = 0.38). There was evidence of substantial statistical heterogeneity (range of I^2^ between 93% and 98% for the 4 interventions, all p < 0.01) for combined ORs across categories. No single study significantly influenced the meta-regression results.

**Table 3:** Random effects meta-regression results.

Variable	Coefficient	SE	Odds Ratio			Z	P-value
			Est.	Lower 95% CI	Upper 95% CI		
Intercept	-1.13	0.31	0.32	0.18	0.59	-3.66	0.0003
Arm							
Control Intervention* (reference)							
FIT	2.26	0.61	9.6	2.89	31.82	3.69	0.0002
PN	1.04	0.42	2.84	1.23	6.49	2.46	0.01
Print	0.63	0.46	1.87	0.76	4.62	1.35	0.18

*Control Intervention denoted the control group in the studies.

### Publication bias analysis

As depicted in a symmetric funnel plot (see S1 Fig), no publication bias was found in this study [88]. Moreover, no evidence of bias was detected by Egger and colleagues’ test (bias = –1.17, SE = 3.18, P = .72) [89].

## Discussion

### Findings overview

The purpose of this systematic review and meta-analysis was to ascertain which interventions were most effective in increasing CRC screening uptake among African-American men. In our qualitative analysis of the extant literature examining the various types of interventions, patient navigation and the distribution of free stool-based test kits, including FIT and iFOBT kits, emerged as the most consistently effective interventions. Print and other educational materials were the most common interventions, but their results were mixed, with some studies reporting increased screening rates compared with controls, while others reported similar or lower screening rates compared with control groups. Through using the Cochrane risk of bias tools to assess the eligible studies in our review, we found that most RCTs failed to provide any details about the blinding of the participants recruitment method, the allocation concealment method, and/or the outcome assessment. Future RCT research should focus on enhancing the research design quality in these specific areas, particularly in the implementation and evaluation stages. In addition, most of the quasi-experimental studies lacked control groups in their study design. Therefore, the ability to make group comparisons among participants was challenging and it was nearly impossible to compare the interventions’ effectiveness. Statistical evidence with both clearly defined controls are needed in future studies.

Heterogeneity of the findings made it challenging to determine which intervention was most effective and should be considered for future studies focused on African-American men as recommended by Kwaan and Jones-Webb [90]. Due to the diversity of settings, geographic regions, interventions employed, and outcomes measured, factors other than the intervention may have influenced CRC screening uptake. For example, most studies were conducted within a medical clinical or hospital. This setting in and of itself may have increased CRC screening uptake by increasing access to other resources (e.g., electronic health records, scheduling services, screening materials).

Further, it should be noted that most of the interventions were implemented by healthcare professionals, including physicians, public health workers (e.g., patient navigators), and the researchers themselves. This may have influenced screening uptake rates as participants may have had an implicit bias toward obtaining CRC screening to please researchers or doctors. Conversely, medical mistrust—a common factor that significantly contributes to delays in healthcare system utilization by African-American men—may have discouraged African American study participants specifically from obtaining CRC screening [3].

### Future directions

Only 2 of 41 studies reviewed (5%) focused exclusively on African-American males [57, 66]. Though each study included some African-American males, researchers rarely examined barriers and enablers specific to this group, which continues to suffer the most from CRC incidence and mortality [1]. The lack of understanding of CRC screening-completion barriers and enablers among African-American men was exacerbated in our study by the lack of distinction in the data reported for race and gender. Studies that segmented data by race or gender did one or the other but not both, leaving the African-American male experience with CRC screening further underrepresented in intervention studies. To achieve the goal of reducing CRC-related inequities among African-American men, health promotion and prevention interventions that centralize cultural identity and cultural empowerment should be developed in order to better capture African-American men’s CRC screening experiences within a culture-specific context and their understanding of those experiences [91, 92].

Our inclusion criteria for the review required a study population that included African-American men, and this specification may have been the cause of the geographic dispersion among the interventions evaluated. The U.S. Census Bureau reports that a majority of the African-American population of the U.S. is concentrated in the Southern and Northeastern regions [93]. Additionally, African Americans experience greater CRC incidence, higher mortality, and lower survival at all stages, when compared to their white counterparts, and several articles mentioned that the geographical locations where interventions were implemented had large populations of African Americans experiencing CRC disparities [3, 19, 49, 59, 78]. However, the majority of the studies (76%) is this review occurred in Eastern and Southern states, with only 25% in Western and Midwestern states. The preponderance of data from the East and South––while helpful for providing insight into regional barriers and enablers to CRC screening uptake––is not equally applicable to all African-American populations. Given geographical variability in diet, culture, and intergenerational attitudes along with the effect of these variables on CRC screening outcomes, more-specific regional information is required to develop effective interventions in understudied areas. Western states, though included in only 12% of the studies evaluated, are home to the nation’s most ethnically diverse populations and currently represent what is projected to be a nationwide shift in the ratio of ethnic-to-nonethnic residents [94, 95]. Specifically, the West encompasses 13 of the 25 most ethnically diverse cities in the nation and the most ethnically diverse state, California, with a census-reported population that is 39% Hispanic-white and 36% non-Hispanic white [94, 96]. As more regions transform to resemble California’s demographic distribution, it is critical that future research includes and emphasizes the barriers and enablers of CRC screening completion in minority-majority areas. More geographically dispersed studies occurring outside of the Southern and Northeastern regions of the U.S. are needed. Moreover, with the unscreened populations in these regions, subgroup differences for those experiencing CRC disparities are yet to be identified.

It is noteworthy that slightly over 75% of the interventions aimed at increasing CRC screening uptake occurred in medical or clinical settings. Although many of these studies did not provide a justification for choosing to provide the intervention in a clinical setting, this decision may have been due to ease of data access, convenience sampling, or community needs. While many of the initiatives to promote CRC screening described in this review took place in healthcare settings and have proved successful, it is likely that individuals who did not seek routine medical care, did not have a regular healthcare provider, or lived with lower socioeconomic status were excluded from the interventions [19]. In particular, the aforementioned scenarios have been demonstrated as potential barriers that prevent African-American men from seeking or obtaining CRC screening [97–100]. Therefore, to reduce the pervasive CRC screening disparities faced by African-American men, it is important for future public health workers, healthcare organizations, patient navigators, researchers, and physicians to consider collaborating to design, evaluate, and implement interventions in non-healthcare settings.

In this review, 24% of the included studies formulated and conducted their CRC intervention programs in churches, local businesses (e.g., barbershops), and other community settings [47, 57, 68, 78, 101]. For example, Holt and colleagues discussed using church-based approaches to promote CRC prevention behaviors through a series of community health advisor–led educational modules [78]. However, results from the educational series suggested that adding spiritual themes did not result in significant behavioral changes among attendees. This might further validate the role of other contributing factors, such as lack of health insurance, lack of access to early-detection screening, medical system mistrust, and socioeconomic disadvantages as mediators influencing early detection screening behavior changes among African-American men, and thus, should be considered alongside the other well-documented barriers to CRC screening [101, 102].

Furthermore, as highlighted in a previous systematic review by Rogers and colleagues, there is a need to better understand the influence of sociocultural determinants that may influence African-American men’s negative responses, reluctance, and apprehension associated with CRC screening [103]. Culturally sensitive community-based interventions among African-American men should be further developed and implemented. However, a few other cautions should be considered by future researchers while designing Federally Qualified Health Center (FQHC) non-clinically–based programs. Maxwell and colleagues noted that it remained challenging to implement and sustain their community-based programs to increase CRC screening among Filipino Americans primarily due to (1) the need for program participants to seek screening through their healthcare providers, (2) lack of funds to sustain the program, and (3) lack of an adequately trained workforce to maintain program activities [104]. Similarly, it is imperative to test the effectiveness of community-based interventions in an environment that supports the sustainable growth of CRC screening promotion programs for African-American men. Moreover, to ensure that African-American men receive the optimal benefits of early detection screening for CRC, researchers must move beyond traditional practice-based settings into community-based locations.

Lastly, from the research team’s observations, cost was a dominant concern or barrier in implementing large-scale CRC screening interventions across the selected studies. Cost-effectiveness strategies require an overall assessment of patient and provider barriers, the navigation system, and other potential inhibitors of CRC screening [105]. Because a significant burden of CRC and observed disparities in CRC screening uptake still exists among African-American men, programs tailored to this population should consider how to effectively enhance knowledge of the benefits of CRC screening, improve access to health care, and elevate the related insurance services [15, 103–105]. In clinical settings, strategies to better utilize patient navigation systems to emphasize the importance of screening and enhance educational outreach for healthcare providers who provide routine care for African-American men could conceivably aid in lowering the cost of promoting CRC screening, particularly among low-income patients [105]. In community settings, interventions that could efficaciously dispel the mistrust and ease the anxiety associated with screening are vital to promoting screening among African-American men [19, 102]. According to Adams and colleagues in a recent systematic review, African-American men often face unique challenges and express substantial fears about medical procedures associated with CRC [106–108]. Adams and colleagues reported that higher mistrust scores correlated with lower CRC screening rates among African-American men in most of the quantitative studies included in their review [96, 106, 109]. In addition, several dominant recurring themes such as “mistrust as a barrier to screening,” “skepticism of provider motives,” and “mistrust of competence and quality of providers/systems” were identified in qualitative studies [74, 106, 110].

Future studies should thoroughly evaluate the effectiveness of different modes of intervention—e.g., patient navigators, telephone outreach, and text messaging. For example, in an effort to explore CRC screening among African-American church members using both qualitative and quantitative methods, the quality of patient-provider communication proved to be the most influential factor in participants’ completion of CRC screening [111]. From the articles included in this review, we can conclude that it is debatable which CRC promotion modes work best for African-American men. However, our meta-analysis results revealed that future interventions utilizing FIT or enhancing patient navigation suited better than traditional methods in increasing CRC screening uptake among this group. Traditional methods included usual care as seen in the control groups of the study, and was significantly inferior to the FIT (*p* = 0.0002) and patient navigation interventions (*p* = 0.01). Print interventions were also secondary juxtaposed to FIT (*p* = 0.001). As recommended in the 2019 CRC screening messaging guidebook, promoting CRC screening via text messaging could be a cost-effective strategy to improve interventions compared with traditional methods (e.g., mailings, printed materials, telephone reminders) [112]. More research and evidence are warranted to identify more cost-beneficial interventions focused on motivating unscreened African-American men to seek recommended CRC screening.

### Limitations

Our study is not without limitations. First, although our publication bias analysis found no such bias, it cannot be ruled out, as studies with negative findings were less likely to be published. Secondly, a significant challenge with our meta-analysis was the heterogeneity of the published data. Most of the studies we reviewed could not be included in the meta-analysis because they did not provide sample or study-outcome data specific to African Americans, especially African-American men, or because the interventions were too dissimilar to combine with other studies. Moreover, since most studies had different inclusion criteria, it was impossible to adjust for a confounder or covariate unless all levels of the covariate were available in all studies. Since this resulted in difficulty teasing out which intervention components were most effective, the meta-analysis results must be interpreted with caution. Next, due to the small sample, aforementioned heterogeneity, and the notion that ‘the best CRC screening test is the one that gets done’, the data captured for the meta-analysis focused on the proportion of participants who completed CRC screening juxtaposed to specific types of screening. On account of the significant difference in the nature of CRC screening modalities, such as colonoscopy vs. FIT, separate analyses (both in future systematic review and meta-analyses) may reveal different results regarding strengthening future interventions for increasing CRC screening completion. Furthermore, since most clinical trials require 2–4 phases—potentially causing minor to major percentage differences for CRC screening uptake based on the effects of different intervention approaches, a more thorough systematic approach that compares these differences as well as conduciveness of different CRC screening modalities in clinical versus community settings may be useful to detect the intervention outcomes. Nevertheless, our findings highlight the lack of consensus in the literature regarding interventions for increasing CRC screening uptake, especially among African-American males. Lastly, although our research team made every effort to ensure our search yielded all applicable data and no publication bias was found in this study, it is possible that some articles were missed and the ability of bias to distort results of future meta-analyses and systematic reviews should be considered.

## Conclusions

In summary, this systematic review and meta-analysis examined the existing evidence for interventions aimed at increasing CRC screening uptake among African-American men. Most of the included studies used approaches such as patient navigation, telephone outreach, targeted brochures, and other multicomponent promotion packages to enhance CRC screening rates. Yet, our findings reflected a dearth of studies unambiguously focused on African-American men. Only 2 of the 41 studies in our review (5%) specifically explored the efficacy of CRC screening-promoting initiatives among African-American men. Since half of the reviewed studies were guided by 1 or multiple conceptual frameworks, a greater number of theory-driven CRC screening interventions are needed. Since studies with the lowest risk of bias employed 4 or fewer interventions, future multicomponent interventions should consider this evidence when designing and implementing CRC screening completion-focused studies among African-American men and other underserved populations. To achieve the National Colorectal Cancer Roundtable’s challenge to attain screening rates of 80% or higher in every community, further study is warranted that considers employing evidence-based, cost-effective, and culture-specific techniques targeting CRC screening completion among African-American men outside of traditional clinic settings.

## Supporting information

S1 Appendix Search strategy syntax. (PDF)

S2 Appendix PRISMA checklist. (PDF)

S1 Fig Begg’s funnel plot with 95% confidence limits. (PDF)
