# Supplementary material for: Interventions for increasing colorectal cancer screening uptake among African-American men: A systematic review and meta-analysis
Source: PLoS One. 2020 Sep 16;15(9):e0238354. doi: 10.1371/journal.pone.0238354 (PMC7494124; doi:10.1371/journal.pone.0238354)
Supplement: S1 Appendix — (PDF) [file pone.0238354.s001.pdf]

1. exp Colorectal Neoplasms/
2. ((colorectal or colon*) adj2 (cancer* or neoplasm* or tumor* or carcinoma*)).ti,ab.
3. or/1-2
4. exp Colonoscopy/
5. exp Colonography, Computed Tomographic/
6. exp Sigmoidoscopy/
7. exp Mass Screening/
8. exp "Early Detection of Cancer"/
9. exp Digital Rectal Examination/
10. ((rectal or colon*) adj1 exam*).ti,ab.
11. (colonoscop* or sigmoidoscop* or colonography or fobt).ti,ab.
12. screen*.ti,ab.
13. or/4-12
14. 3 and 13
15. (crc adj1 screen*).ti,ab.
16. 14 or 15
17. exp African American/
18. (african american* or black*).ti,ab.
19. 17 or 18
20. exp MEN/
21. (men or male*).ti,ab.
22. or/20-21
23. 16 and 19 and 22
