# Supplementary figures and images for: Interventions for increasing colorectal cancer screening uptake among African-American men: A systematic review and meta-analysis
Source: PLoS One. 2020 Sep 16;15(9):e0238354. doi: 10.1371/journal.pone.0238354 (PMC7494124; doi:10.1371/journal.pone.0238354)

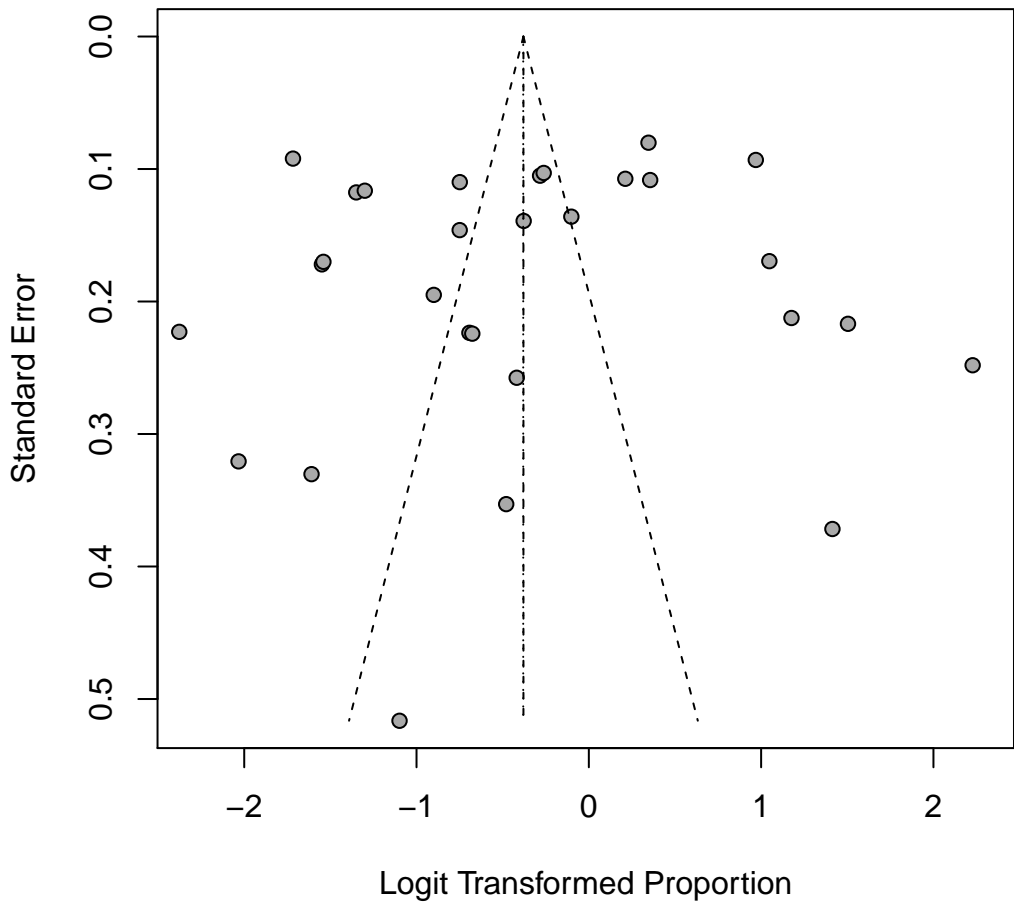

Supplement: S1 Fig — (PDF) [file pone.0238354.s003.pdf]
